# Human Sexuality

**Published:** 1906-12

**Authors:** 


**Human Sexuality.** A Medico-Literary Treatise on the Laws, Anomalies, and Relations of Sex, with special reference to contrary sexual desire. By **J. Richardson Parke**, M.D., (Late Acting Assistant Surgeon, U. S. Army). Octavo, 476 pages. Professional Publishing Company, Philadelphia. 1906. (Price, $3.00 net).

This work is a monument of industry. The author has studied the subject as it exists in lands civilised and uncivilised and presents to the reader much that is new as well as very much that is old and threadbare. The rehash of personal experience of perverts is given a prominent place in the volume. The reader who looks for variety of sexual customs will find it here described. A very complete list of authorities on the subject is given and we can recommend the book as being thorough and scientific. We cannot say, however, that it is helpful. Not a word do we find which can be of the least benefit to the afflicted. Really, we have had enough of printed experience of sexual custom and sexual crime. On the other hand, we have not had sufficient help in the treatment of these unfortunates.

